# Triple combination of vemurafenib, cobimetinib, and atezolizumab in real clinical practice in the Russian Federation: results of the A1 cohort of the ISABELLA study

**DOI:** 10.3389/fonc.2024.1395378

**Published:** 2024-10-14

**Authors:** Igor V. Samoylenko, Yulia M. Kolontareva, Ekaterina V. Kogay, Natalia V. Zhukova, Igor A. Utyashev, Mikhail E. Ivannikov, Konstantin V. Menshikov, Maxim V. Zinkevich, Kristina V. Orlova, Yulia V. Vakhabova, Mikhail V. Volkonsky, Natalia A. Beliaeva, Ivan I. Butkov, Elena V. Karabina, Tatyana L. Moskovkina, Kseniya A. Moshkova, Olga V. Plishkina, Vitaliy D. Sychev, Oxana S. Cheplukhova, Vera V. Chernova, Alexandr N. Yurchenkov, Ksenia G. Babina, Nikita A. Savelov, Lev V. Demidov

**Affiliations:** ^1^ Skin tumors department, NN Blokhin National Medical Research Center of Oncology, Moscow, Russia; ^2^ The Russian Melanoma Professional Association (Melanoma.PRO), Moscow, Russia; ^3^ Hoffman-La Roche, Scientific Health Chapter, Moscow, Russia; ^4^ St. Petersburg City Clinical Oncology Dispensary, St. Petersburg, Russia; ^5^ Hadassah Clinic, Moscow, Russia; ^6^ Moscow Regional Clinical Oncological Dispensary, Balashikha, Russia; ^7^ Ufa Republican Clinical Oncological Dispensary of the Ministry of Health Republic of Bashkortostan, Ufa, Russia; ^8^ Leningrad Regional Clinical Oncological Dispensary, St. Petersburg, Russia; ^9^ European Medical Center, Moscow, Russia; ^10^ Moscow City Oncological Hospital No.62, Moscow, Russia; ^11^ Orenburg Regional Clinical Oncological Dispensary, Orenburg, Russia; ^12^ Medical Center Medical City, Tyumen, Russia; ^13^ Tula Regional Clinical Oncological Dispensary, Tula, Russia; ^14^ Kurgan Regional Oncological Dispensary, Kurgan, Russia; ^15^ Nizhny Novgorod Regional Clinical Oncological Dispensary, Nizhny Novgorod, Russia; ^16^ Kirov Center of Oncology and Medical Radiology, Kirov, Russia; ^17^ Tambov Regional Clinical Oncological Dispensary, Tambov, Russia; ^18^ Murmansk Oncological Dispensary, Murmansk, Russia; ^19^ Ryazan Clinical Oncological Dispensary, Ryazan, Russia; ^20^ Volgograd Regional Clinical Oncological Dispensary, Volgograd, Russia

**Keywords:** metastatic melanoma, BRAF-mutant, triple combination, brain metastases, atezolizumab, vemurafenib, cobimetinib

## Abstract

**Background:**

Among several treatment options for BRAF-mutant metastatic melanoma, a combination of BRAF inhibitor, MEK inhibitor, and anti-PDL1 antibody seems to be a new emergent approach recently registered in the Russian Federation. It is still not clear which patient population benefits more from this simultaneous use of three drugs instead of its sequencing.

**Aim:**

This study aimed to evaluate patients’ characteristics treated in real practice in 14 Russian regions by triple combination and to analyze their outcomes depending on biomarkers (PD-L1 expression).

**Methods:**

This was a part (cohort A1) of a prospective non-interventional study of clinical outcomes and biomarkers in patients with skin melanoma. Patients were included in cohort A1 if combination treatment with vemurafenib (vem) + cobimetinib (cobi) + atezolizumab (atezo) was initiated no earlier than 12 weeks (84 days) prior to written informed consent to participate in this study. The index event was the initiation of therapy with all three drugs vem + cobi + atezo (i.e., triple combination). The primary efficacy endpoint of the study was the 24-month overall survival (OS), defined as the time from the index date to the date of death from any cause. If the patient did not experience an event, the OS will be censored at the date of the last contact. Objective response rate (ORR), duration of response (DoR), and progression-free survival (PFS) in the Intention to treat (ITT) population, in biomarker positive population, and in population with brain metastases were also evaluated. Quality of life questionnaires were pre-planned by protocol if it was a part of routine practice. Adverse events were also collected.

**Results:**

Between March 2021 and May 2023, 59 patients were enrolled in 19 centers from 14 regions of Russia. Thirty-one of 59 (52.4%) patients had central nervous system metastases, and 18 of 31 (58.4%) were symptomatic. Forty of 59 patients (68%) received the triple combination as the first-line treatment. The median follow-up period was 16.83 [95% confidence interval (CI) 13.8–19.8] months. The mean duration of therapy with this regimen was 9.95 months (95% CI 7.48–13.8). ORR was 55.1%; progression as the best outcome was seen in 16.3%. The median DoR was 12.95 months (95% CI 11.0–14.8 months), with a median of 20.3 months (95% CI 9.1–31.5 months) when triple therapy was administered in the first-line treatment. In patients with brain metastases (*N* = 31), ORR was 45.1%; the median DoR was 12.95 (95% CI 11.0–14.8 months). The median PFS in the entire population was 13.6 months (95% CI 8.6–18.6); the 24-month PFS was 22%. The estimated median OS in the entire population was 15.8 months (95% CI NA); 24-month OS was 45% (95% CI 0.32–0.64). In multivariate Cox regression model, biomarkers of interest [lactate dehydrogenase, Programmed cell death ligand-1 (PD-L1)] did not have statistically significant impact on PFS, OS, or DoR probably due to high data missing rate. No unexpected adverse events were reported. Grades 3–4 AEs were seen in 23 of 59 patients (38%) with most common were skin and liver toxicity.

**Conclusion:**

Triple combination of atezolizumab, vemurafenib, and cobimetinib had proven its efficacy and tolerability in real settings. No impact of potential predictive biomarkers was seen (NCT05402059).

## 1 Introduction

Despite its relatively low incidence (approximately 12,000 new cases per year in the Russian Federation), melanoma can be a difficult-to-treat neoplasm with a high recurrence rate, even among patients who have remained in remission for a long time (1, 2). Patients with metastatic melanoma and a mutation in the BRAF gene can be both treated with immunotherapy, including anti-CTLA4+anti-PD1 combination therapy or targeted therapy with BRAF and MEK inhibitors. The data of most studies demonstrate that the use of combined targeting therapy has a time-limited benefit in at least 60%–75% of patients; therefore, this type of treatment is considered as optimal mainly in patients who are not candidates for immunotherapy (1, 3, 4, 11). Also in 2022, a combination of targeted and immunotherapy—vemurafenib, cobimetinib, and atezolizumab—was registered in the Russian Federation, which showed its benefit over combined vemurafenib and cobimetinib arm in the IMspire150 phase III study (5). Triple combination of vemurafenib, cobimetinib, and atezolizumab has also shown encouraging results in patients with symptomatic brain metastases in the phase II Tricotel study (6). It was also been shown that triple combination significantly improves progression-free survival (PFS) compared to BRAFi+MEKi alone specifically in patients with PD-L1 expression >1%: median PFS in this subgroup in vemurafenib and cobimetinib group was 11.4 months (158 patients) and in the vemurafenib, cobimetinib, and atezolizumab group 14.8 months (160 patients), with a hazard ratio of 0.80 (0.60–1.06) (6). A very similar results were obtained in another phase III study, COMBI-I: in patients with PD-L1 ≥1%, the risk ratio was 0.76 (0.54–1.07) in favor to triple therapy (dabrafenib + trametinib + spartalizumab vs. combined targeted therapy), while in negative group PD-L1 <1% the risk ration was higher 0.84 (0.60–1.18) (7). Despite the lack of statistical significance, the results seem to be very interesting.

Moreover, secondary endpoints, such as duration of response (DoR) or even PFS in the group with normal LDH levels, were significantly better on triple therapy only in patients with PD-L1 expression levels >1% (22.7 vs. 12.9, OR: 0.67) (7). If these assumptions are confirmed and proved, the corresponding biomarker could be used to select candidates for triple therapy, which would be eminently reasonable given the potential toxicity and the high cost of this treatment regimen. Given the increasing number of possible treatment options for metastatic melanoma patients in first-line therapy (aPD1+aCTLA4, or aPD1, or BRAFi+MEKi, or aPD1/aPDL1+BRAFi+MEKi), we believe that the use of a biomarker, such as PD-L1 expression level, could be very useful. However, the routine use of this biomarker is significantly hampered by the availability of different test systems with different positivity thresholds.

Because routine drug administration, including dosing, treatment interruption, and early treatment discontinuation, in clinical practice may differ from the procedures used in clinical trials, post-registration “real-world” data are important to quantify the feasibility, acceptability, and practical considerations for prescribing targeted and immunotherapy. Therefore, it is of great interest to the clinical and research communities to evaluate patient and treatment choices used in the daily practice of cancer centers in Russia.

With these in mind, we conducted an observational non-interventional study to explore the usage of triple combination in real-world practice without restrictions on lines of therapy and disease burden.

We publish so far only the results of the A1 cohort of the larger observational study MelPRO-0921 (Isabella). The aim of this study is to evaluate clinical outcomes in patients with stages III–IV skin melanoma in real clinical practice in the context of different levels of PD-L1 expression in the tumor and other potential biomarkers. In addition, it is of interest to gain insights into real-world data regarding the quality of life of melanoma patients treated with targeted and immunotherapy for metastatic disease.

## 2 Materials and methods

From September 2021, marked by the first patient’s first visit, to September 2023, including 6 months after the last patient’s first visit (LPFV + 6 months), a prospective, non-interventional study was conducted in cohort A1. This study assessed clinical outcomes and biomarkers in patients with stages III–IV unresectable skin melanoma within real-world clinical settings. Patient recruitment for additional cohorts is currently active, with their respective analyses to be detailed in future publications.

Eligibility for cohort A1 required patients to have histologically confirmed stage IV metastatic skin melanoma or stage IIIC/D unresectable melanoma, including cases of skin melanoma metastases with unknown primary origin. Additionally, patients must have a confirmed BRAF mutation and be slated by their treating physician to begin treatment with the combination of vemurafenib, cobimetinib, and atezolizumab (AVC) before entering the study. Inclusion in the study mandated written informed consent from all participants. The study protocol received approval from the institutional review board.

Initiation of treatment with the combination therapy consisting of vemurafenib, cobimetinib, and atezolizumab was mandated to commence no more than 12 weeks (84 days) prior to obtaining written informed consent from participants in this study. All individuals were required to have an Eastern Cooperative Oncology Group (ECOG) performance status of ≤3 at the time of the index event and be aged over 18 years.

The index event for cohort A1 was defined as the commencement of therapy with the triple combination of vemurafenib, cobimetinib, and atezolizumab. Patient visits to the research centers were conducted according to routine clinical practice, with observational visits scheduled every 3–4 months as delineated in current clinical guidelines (cr.rosminzdrav.ru). Standard clinical procedures and examinations were carried out in accordance with these guidelines and based on physician discretion.

Data collection involved standard management practices for patients under routine clinical conditions during their visits to the research centers. For patients either scheduled for or having already received atezolizumab, an archival paraffin-embedded tumor block was requested for PD-L1 expression analysis, conducted using the Dako 22C3 platform in the central laboratory.

The primary efficacy endpoint for the study is the 24-month overall survival (OS), defined as the interval from the index date to death from any cause. In instances where no event occurred, the OS data were censored on the date of last contact with the patient.

As the study does not entail testing a predefined statistical hypothesis, calculations related to sample size and statistical power are deemed inapplicable. Consequently, the sample size is determined based on a consensus among protocol authors, aiming to enroll a minimum of 50 patients within the stipulated recruitment period.

This study is registered under the identifier NCT05402059 on Clinicaltrials.gov.

## 3 Results

Between 15 March 2021 and 10 May 2023, the A1 cohort included 59 patients from 19 centers across 14 regions of Russia. The median follow-up period was 16.8 months (95% CI 13.8–19.8 months). The mean duration of therapy with this regimen was 10.0 months (95% CI 7.48–12.42 months).

The baseline characteristics of the patients are summarized in **Table 1**.

**Table 1:** Baseline characteristics of patients.

Indicator	*N*	%
**Total patients**	59	100
Sex		
Men	27	45.8
Age, median (min–max): 50.3 years (20.5–85.6)		
ECOG		
** 0**	21	35.6
1	30	50.9
2	6	10.2
3	2	3.4
Stage at study entry:		
III (C/D) unresectable	1	1.7
IV M1a	8	13.6
IV M1b	4	6.8
IV M1c	15	25.4
IV M1d	31	52.5
Total number of metastasis sites		
** 3 or more**	47	79.6
less than 3	10	17.0
No data	2	3.4
Localization of metastases		
** Skin/regional lymph node/soft tissue**	40	67.8
Liver	12	20.3
Bones	17	28.8
Lungs	35	59.3
other sites	30	50.9
CNS	31	52.5
Type of CNS metastases (among all CNS metastases)		
** Symptomatic**	18	58.1
Asymptomatic	9	29.0
After stereotactic radiotherapy	3	9.7
No data	1	3.2
Tumor burden (estimated):		
0…5 cm	22	37.3
6…10 cm	12	20.3
10…15 cm	9	15.25
over 15 cm	14	23.7
No data	2	3.4
Preferred imaging type:		
CT with IV contrast	11	18.6
PET-CT with 18 FDG and IV contrast	46	78.0
LDH level		
<UL	20	34.0
1 to 2 UL	10	17.0
>2 UL	2	3.4
No data	27	45.8
PD-L1 expression, %		
PD-L1 < 1	20	58.8
PD-L1 > 1	3	8.8
PD-L1 > 5	2	5.9
PD-L1 > 10	4	11.8
No data	5	14.7
BRAF V600 mutation type		
V600E	56	94.9
V600K	2	3.4
V600D	4	6.8
Other	1	1.7
Line of therapy		
1st line	40	67.8
2nd line	7	11.9
3rd line	7	11.9
>3 lines.	5	8.5
Рrior adjuvant therapy	12	20.3
BRAFi+MEKi	2	3.4
IFN alfa	5	8.5
aPD1	4	6.9
Chemo	1	1.7
Prior treatment for metastatic disease	19	32.2
BRAFi ± MEKi	11	18.6
aPD1+aCTLA4	3	5.1
aPD1	4	6.8
Chemo	1	1.7

In our study cohort, central nervous system (CNS) metastases were detected in 31 patients (52.4%), with 18 of these individuals (58.4% of those with CNS metastases) presenting neurological symptoms. These symptoms ranged from generalized cerebral manifestations such as headaches and dizziness to more specific neurological impairments, including meningeal or focal symptoms, necessitating the administration of glucocorticosteroids and/or anticonvulsants. Unfortunately, it was not feasible to gather data regarding the dosages of glucocorticosteroids prior to the initiation of treatment or during the period of combined immune-targeted therapy.

It should also be noted that lactate dehydrogenase (LDH) levels were unreported in 27 patients (46%), which likely indicates that these tests were not conducted. The inconsistent measurement of LDH levels persists as a common issue in numerous oncological centers across Russia.

Additionally, a substantial proportion of the cohort, approximately 68% (40 patients), received triple combination therapy as their initial treatment strategy. Patients were classified as receiving second-line or subsequent treatment if they had previously undergone therapy for unresectable or metastatic melanoma. Notably, adjuvant therapy was not considered as a line of treatment in this classification. Information on the efficacy of this therapy regimen is summarized in **Table 2**.

**Table 2:** Response rates and response duration in patients treated with atezolizumab, vemurafenib, and cobimetinib.

Best response to therapy	*N* = 59	Minimum registration time, months	Median time of onset, months	Maximum registration time, months
Complete response (CR)	5 (8.5)	3.3	4.2	10.7
Partial response (PR)	26 (44.1)	1.6	3.4	11.7
Stabilization	13 (22.0)	1.7	3.07	5.4
Progression	12 (20.3)	1.9	3.02	4.8
Unable to evaluate	3 (5.1)			

In a cohort analysis where 49 patients were assessed for therapy effectiveness, 27 exhibited treatment responses, resulting in an objective response rate (ORR) of 52.6%. Progression was reported as the best outcome in 12 of these patients (20.3%). The median duration of response to therapy was 13 months (95% CI 11.0–14.8 months), with a median of 20.3 months (95% CI 9.1–31.5 months) when AVC therapy was administered in the first line.

**Figure 1:**
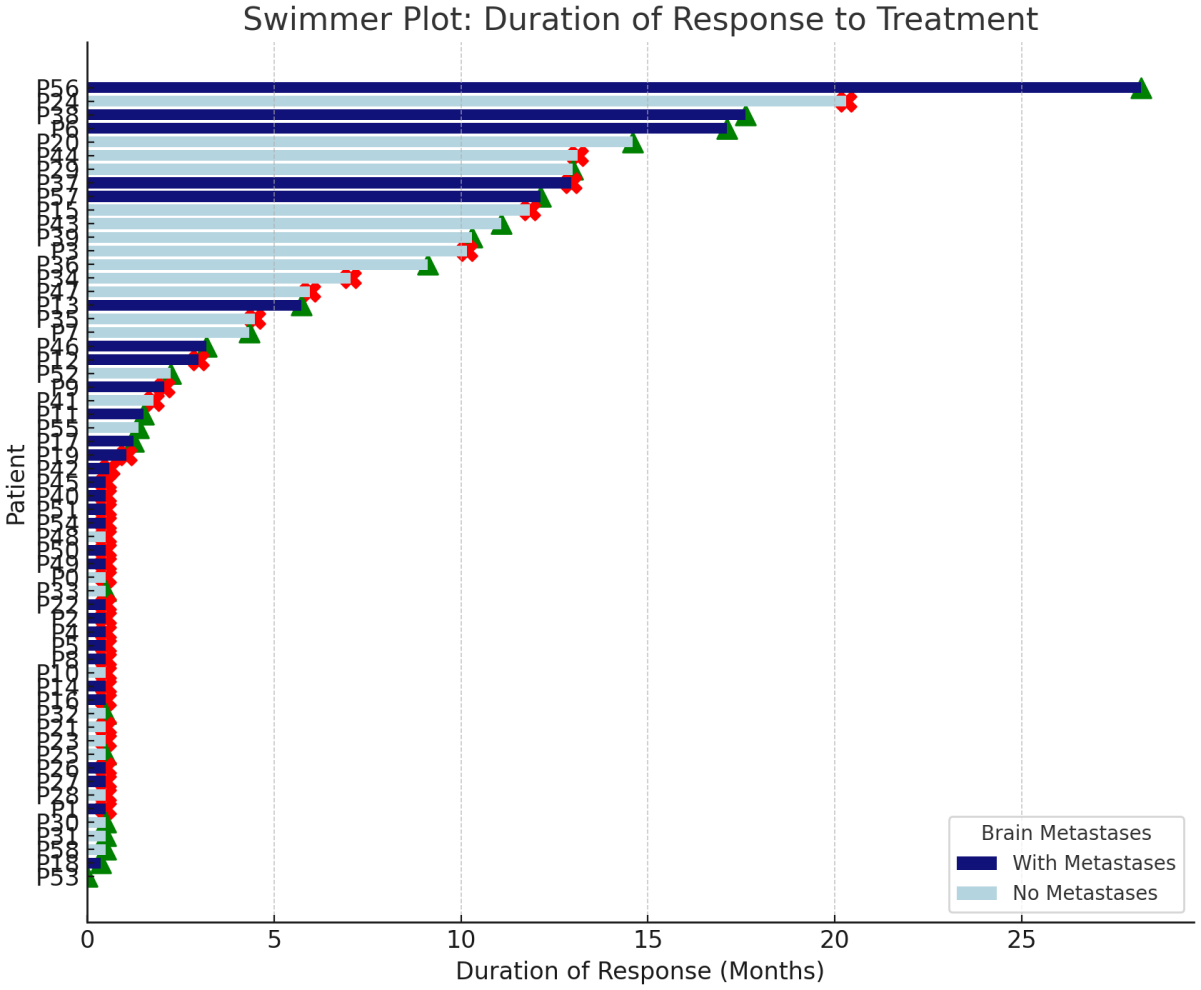
Individualized duration of response to therapy in patients with and without brain metastases. Green triangles indicate ongoing response; red crosses indicate onset of disease progression.

In the first-line settings, 4 (9.3%) patients achieved a complete response (CR), 19 (44.2%) achieved a partial response (PR), 10 (23.3%) maintained stable disease (SD), 8 (18.6%) experienced disease progression as the best overall response, and 2 (4.7%) were not evaluable due to inadequate imaging methods. In subsequent lines of therapy, one (6.3%) patient achieved CR, seven (43.8%) achieved PR, three (18.8%) maintained SD, four (25%) experienced disease progression as the best overall response, and response for one patient (6.3%) was not evaluable due to relocation and hospital change with no additional information provided by the study site. Individualized duration of response to therapy is presented in **Figure 1**.

In a cohort of 31 patients with brain metastases, a therapeutic response was observed in 45.1% of cases. The median duration of response among symptomatic individuals was 12.95 months (95% CI 11.0–14.8 months).

**Figure 2:**
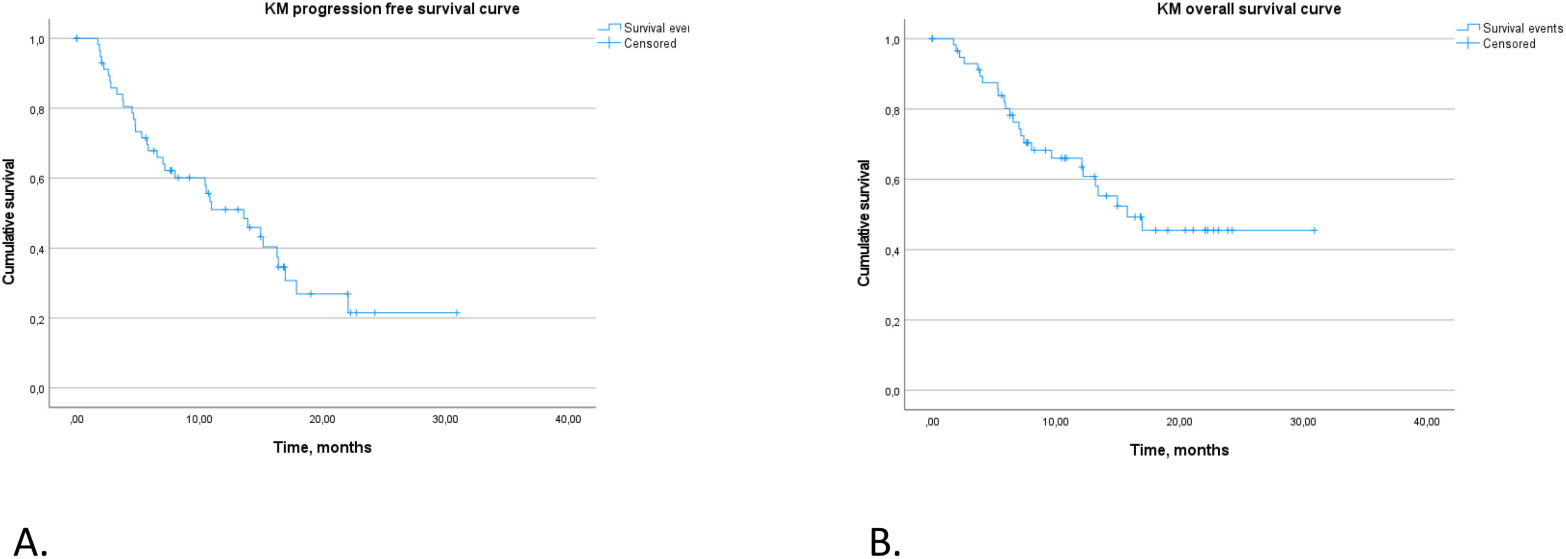
Progression-free survival **(A)** and overall survival **(B)** for the entire population.

For the entire study population, the median PFS was recorded at 13.6 months (95% CI 8.6–18.6 months). The rate of PFS at 12 months was 51% (95% CI 39%–66.8%), decreasing to 22% at 24 months, as illustrated in **Figure 2A**.

The estimated median OS across the cohort was 15.8 months, with the 95% CI not calculated due to the limited number of events. The 12-month OS rate was 66% (95% CI 0.54–0.8), and the 24-month OS rate was 45% (95% CI 0.32–0.64), also detailed in **Figure 2B**.

**Figure 3:**
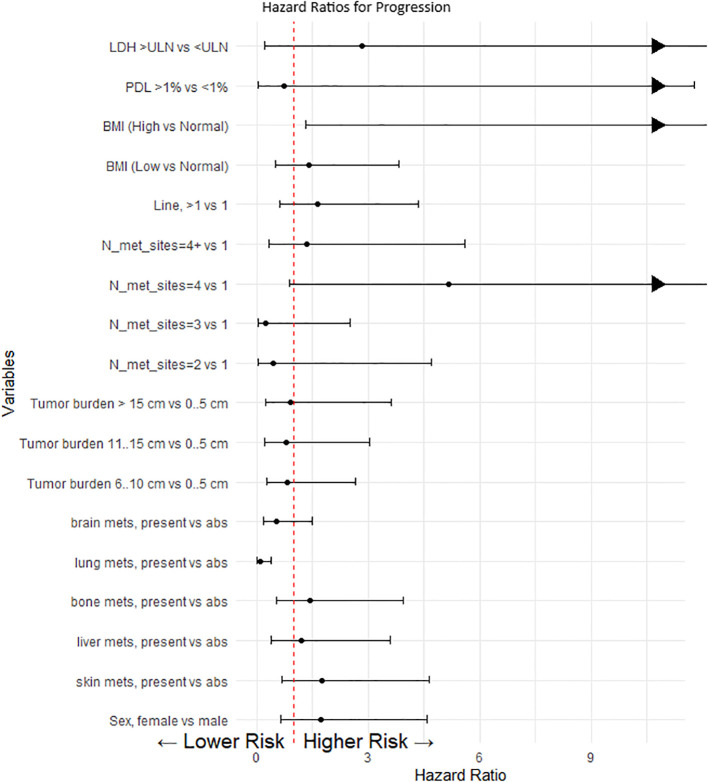
Cox proportional model and multivariate analysis for progression. This forest plot visualizes the hazard ratios (HR) for various clinical and demographic factors affecting progression-free survival in our patient cohort. Each point represents the hazard ratio for a specific category compared to a reference category, with the horizontal lines indicating the 95% confidence intervals (CI). A value of 1 (marked by a dashed red line) indicates no effect. Values greater than 1 suggest a higher risk of progression, whereas values less than 1 suggest a lower risk. When the upper limit of the confidence interval exceeds the scale (e.g., greater than 10), an arrow is used to indicate that the effect extends beyond the plotted range.

The presence of lung metastases and elevated body mass index (BMI) were identified as significant determinants of PFS, suggesting increased risks associated with the absence of lung metastases and higher BMI values (refer to **Figure 3**). Other variables, including disease prevalence, LDH levels, and PD-L1 expression, failed to reach statistical significance in impacting PFS, likely attributable to the limited statistical power of the study (see **Figure 3**).

**Figure 4:**
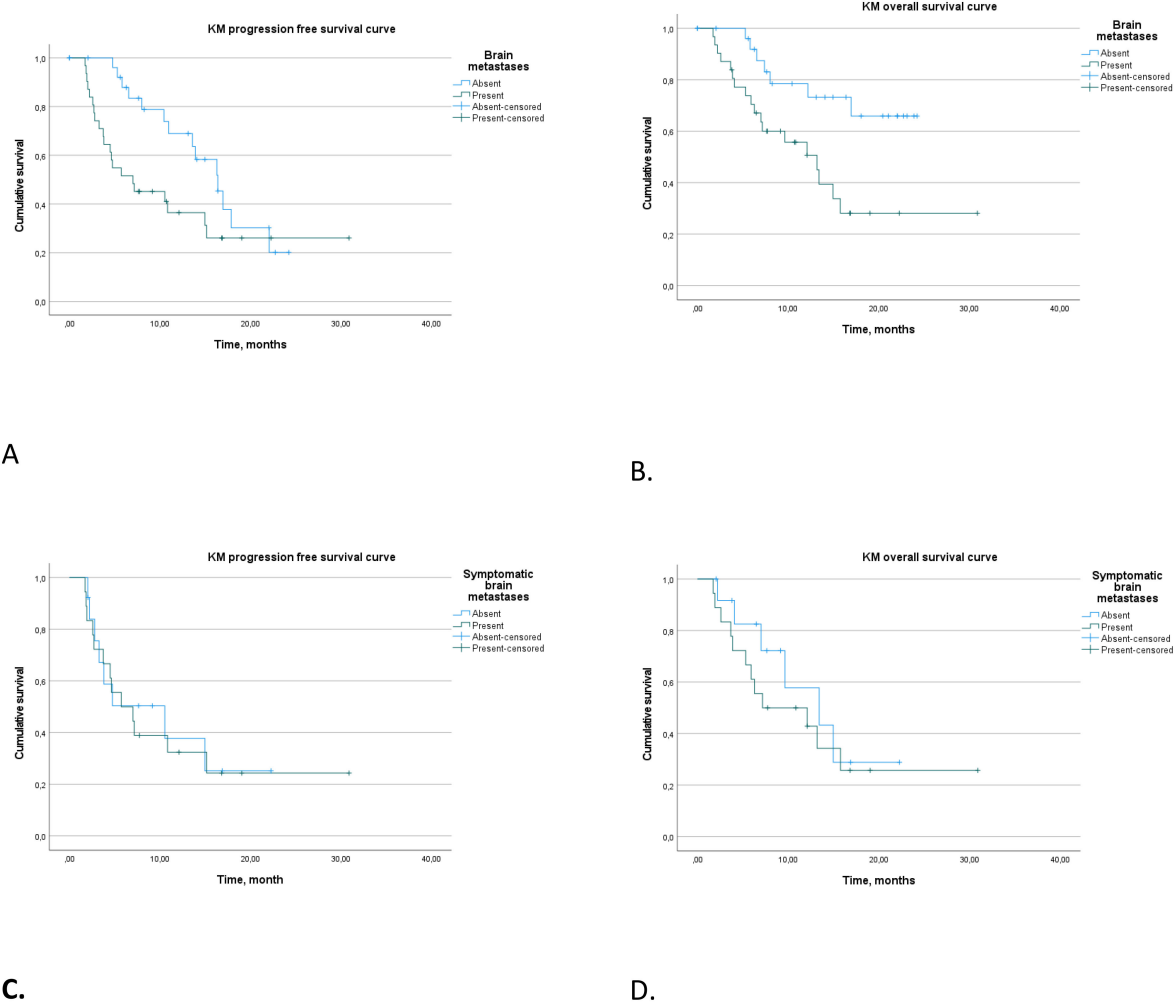
Progression-free survival **(A)** and overall survival **(B)** in patients with and without brain metastases. Progression-free survival **(C)** and overall survival **(D)** in patients with symptomatic and asymptomatic brain metastases.

In our analysis, we investigated PFS and OS outcomes in patients with and without brain metastases, with a focus on symptomatic versus asymptomatic presentations (**Figures 4A–D**).

Patients with brain metastases demonstrated a median PFS of 7.1 months (95% CI 0.9–13.1), significantly shorter than the 16.4 months (95% CI 13.0–19.8) observed in patients without brain metastases (log rank *p* = 0.046). Patients with symptomatic brain metastases did not show a statistical difference in median PFS compared to asymptomatic cases (*p* > 0.5).

Further analysis of patients with brain metastases who received triple therapy in the first-line setting revealed median PFS of 5.7 months (95% CI 2.6–8.9 months) for symptomatic cases and 10.5 months (95% CI 0–22.4 months) for asymptomatic cases (*p* > 0.5).

In terms of OS, patients with brain metastases had a median OS of 13.2 months (95% CI 8.6–17.8), with those receiving first-line treatment showing a slightly extended median OS of 15.4 months (95% CI 5.0–19.1 months). Patients without brain metastases did not reach a median OS (log rank *p* = 0.008). Symptomatic and asymptomatic brain metastases did not differ significantly in terms of median OS (*p* > 0.5).

Our findings provide insights into the differential impact of brain metastases and their symptomatic status on survival outcomes, as well as the potential influence of treatment modalities on these outcomes.

In our study of patients with brain metastases, the treatment response rate was 45.1%, irrespective of intra- or extracranial localization, with all responses being partial. Notably, disease progression was the primary type of response in 11 patients (35.4%) (see **Table 3**).

**Table 3:** Best objective response in patients with brain metastases.

	Brain metastases		Total *N* (%)
	Asymptomatic	Symptomatic	
Complete responses	1 (7.7)	0	1 (3.2)
Partial responses	5 (38.5)	9 (50)	14 (45.2)
Stable disease	2 (15.4)	3 (16.7)	5 (16.1)
Progressive disease	5 (38.5)	6(33.3)	11 (35.5)
Total	13	18	31
Duration of response, median (months)	Not reached	12.9	Not reached

Regarding the temporal aspect of treatment response, patients with symptomatic brain metastases exhibited a median duration of response of 12.9 months (95% CI 0–32 months). In contrast, the median duration of response was not reached in patients with asymptomatic brain metastases. These findings underscore the pivotal role of symptomatic presentation in influencing both treatment response rate and prognostic trajectories in the context of brain metastases.

In this study, we also analyzed the effect of the treatment line on PFS for patients receiving AVC combination therapy. The results showed that the median PFS for those starting treatment in the first line was 11.0 months, with a 95% CI of 7.4–14.6 months. For patients treated in the second or subsequent lines, the median PFS was 15.0 months, with a 95% CI of 4.1–25.8 months. However, these differences were not statistically significant (*p* > 0.5).

Similarly, the median OS was not reached for patients who received first-line therapy, indicating that survival extended beyond the study period. In contrast, for those treated in later lines, the median OS was 15.0 months (95% CI 4.1–25.8 months), with no significant difference observed (*p* > 0.5). This data suggests that the timing of treatment initiation does not significantly affect survival outcomes within the study parameters.

Adverse events were reported in 56 of 59 patients (95%) with grades 3–4 AEs seen in 23 of 59 patients (38%) (**Table 4**). Skin toxicity was the most common adverse event, affecting 57% of patients, including severe (grades 3–4) cases in 6.7% (four cases). Hepatotoxicity was noted in 25% of patients, with severe cases (grades 3–4) constituting 17%. Other reported events included anemia and arthralgia, each observed in 10% of patients, with severe anemia in 3% (two cases). Photosensitization was also reported in 10% of the cohort. Less frequent adverse events included pyrexia, diarrhea, hypertension, and weakness, each occurring in 5%–8% of patients, while severe tonsillitis (grades 3–4) was observed in 3%. In summary, the spectrum of adverse events was consistent with known profiles, with no new adverse events detected.

**Table 4:** Adverse events in patients on triple combination AVC.

Adverse events	Grades 1–2	Grades 3–4	Total	Total %	Grades 3–4, %
skin toxicity	30	4	34	58%	7%
hepatotoxicity	5	10	15	25%	17%
anemia	4	2	6	10%	3%
arthralgia	6	0	6	10%	0%
photosensitization	6	0	6	10%	0%
pyrexia	5	0	5	8%	0%
diarrhea	3	1	4	7%	2%
hypertension	2	1	3	5%	2%
weakness	3	0	3	5%	0%
alopecia	2	0	2	3%	0%
sore throat	0	2	2	3%	3%
hypothyroidism	2	0	2	3%	0%
headache	2	0	2	3%	0%
colitis	1	1	2	3%	2%
ophthalmic toxicity	2	0	2	3%	0%
ascites	1	0	1	2%	0%
leukopenia	0	1	1	2%	2%
limb swelling	1	0	1	2%	0%
pneumonia	0	1	1	2%	2%
pulmonitis	1	0	1	2%	0%
vomiting	1	0	1	2%	0%
oral dryness	1	0	1	2%	0%
tachycardia	1	0	1	2%	0%
nausea	1	0	1	2%	0%
thrombocytopenia	1	0	1	2%	0%
lymph node sarcoidosis	1	0	1	2%	0%

Quality of life data were not reported due to extremely low return rate of questionnaires.

## 4 Discussion

It is now evident that, for asymptomatic BRAF-mutant melanoma patients, the combination of ipilimumab and nivolumab is the most effective treatment option. In the BRAF V600 positive population, this combination extended median PFS to 16.8 months, with 6.5-year OS data indicating the highest rates among all explored treatment options at 57% (median OS not reached) (12).

The DREAMSeq study also demonstrated a significantly better OS benefit for patients who started with ipilimumab and nivolumab compared to those who received dabrafenib and trametinib (3).

However, there remains a subset of patients who require the rapid action of the BRAFi+MEKi combination. The DREAMSeq study highlighted that approximately 15% of patients died within the first 6 months and did not have the opportunity to benefit from BRAFi+MEKi therapy (3). This raises the question: could the integration of aPD1/aPDL1 inhibitors with BRAFi+MEKi potentially enhance survival outcomes for patients who are unsuitable candidates for combined immunotherapy? Additionally, which specific patient populations could be selected for the triple combination therapy in real-world clinical practice?

In the IMspire 150 trial, the addition of atezolizumab to the vemurafenib and cobimetinib combination significantly improved PFS, the primary endpoint. Other endpoints, such as duration of response and OS, also were beneficial in the triple therapy arm compared to the double arm, indicating the potential advantage of adding PD-L1 to targeted therapy for metastatic melanoma patients (5).

For metastatic melanoma with CNS disease, systemic treatment efficacy was explored in several studies. The combination of ipilimumab and nivolumab in patients without neurological symptoms showed a 55% intracranial objective response rate (icORR) with median duration of response (mDOR) not reached (CHECKMATE-204 study). However, in patients with neurological symptoms, the efficacy was lower with a 17% icORR. In these patients, targeted therapy appeared more effective, with the dabrafenib and cobimetinib combination achieving a 59% icORR and a 4.5-month mDOR, similar to the effect in asymptomatic patients (58% icORR and 6.5-month mDOR) in the COMBI-MB trial (10, 13).

The TRICOTEL trial examined a triple combination of targeted therapy with immunotherapy in BRAF V600 mutation-positive melanoma patients with CNS metastases. Among the 65 patients, 26 were symptomatic. The outcomes showed a 52% overall response rate with a 42% intracranial ORR, a median overall response duration of 7.4 months, and a median overall PFS of 5.5 months on the triple therapy (atezolizumab + vemurafenib + cobimetinib) (6, 8).

In our study, we investigated the use of the described triple combination in a real-world setting, not limited to the first line and including patients with brain disease. The median PFS in the entire population was 13.6 months, close to the IMspire 150 study (15.6 months) (5), though other efficacy endpoints appeared lower due to the mentioned reasons.

In a subgroup of patients who received triple therapy as first-line treatment (*n* = 40), the median PFS was 11.0 months, and the median OS was not reached. The limitation for OS was due to a small number of events, not reaching 50% at the time of data cutoff.

In our observation, we saw the expected effect of the immune component prolonging the response to treatment. The median duration of response for the entire first-line population was 20.3 months, close to IMspire 150 trial results (21.6 months) and numerically more than in the Keynote-022 study (18.7 months) (9).

We also examined a cohort of patients with CNS metastasis, a population with poor prognosis and high unmet clinical need. In the TRICOTEL study, tapering or discontinuing corticosteroids in patients with symptomatic CNS metastases after a short run-in period with vemurafenib + cobimetinib allowed patients to benefit from subsequent immunotherapy. In our study, the overall response rate (45.1%) and median PFS (7.1 months) in patients with CNS looked little bit lower than in the TRICOTEL study (54% and 5.5 months correspondingly) (6, 8).

For patients with symptomatic metastases, the median PFS was 5.7 months compared to 7.2 months in the TRICOTEL study. For asymptomatic metastases, the median PFS was 10.5 months compared to 7.2 months in the TRICOTEL study (6, 8). It is known that ICI shows less efficacy in patients with CNS neurological symptoms, as seen in the CheckMate 204 study where the ipilimumab and nivolumab combination showed a median PFS of 1.2 months in patients with neurological symptoms and was not reached in asymptomatic patients (13).

In our study, the median duration of response in symptomatic CNS patients was 12.95 months, higher than the 10.2 months in the TRICOTEL study (9). The most prominent duration of response was shown by one patient with symptomatic CNS metastases, who achieved a partial response lasting 27 months and ongoing, despite experiencing adverse events resolved with symptomatic treatment.

The driven hypothesis of PD-L1 expression and LDH level’s influence on outcomes was not supported in our observation, possibly due to a high number of missing PD-L1 and LDH test results. Multivariate regression analysis showed that only the localization of metastases, normal BMI, and the number of metastasis sites significantly affected PFS.

The toxicity profile in our real-world observation was close to the IMspire150 trial, with 95% of patients experiencing adverse events, and skin adverse events and hepatotoxicity being the most common. The lower number of grades 3–4 adverse events might be explained by underreporting due to the observational study design.

Overall, our real-world experience contributes significantly to existing clinical evidence on the efficacy of triplet regimens in melanoma treatment, offering a potential alternative for selected patient groups such as those with symptomatic brain metastases. These findings highlight the need for further clinical trials and comprehensive biomarker analyses to optimize and personalize treatment plans for improved therapeutic outcomes.

## 5 Conclusions

Our research confirmed the reproducibility of phase 3 study outcomes in real-world settings, encompassing a patient cohort characterized by adverse prognostic indicators, including non-first-line therapy recipients, high disease prevalence, and individuals with brain metastases. The study faced limitations in assessing the correlation with a probability biomarker due to a substantial shortfall in the available sample size for analysis. In conclusion, while the use of triplet regimens in melanoma represents a promising therapeutic strategy, especially for challenging cases such as symptomatic brain metastases, extensive clinical research, and sophisticated biomarker-driven approaches are essential to fully understand and utilize the potential of these treatment regimens in tailored patient care.

## Data Availability

The raw data supporting the conclusions of this article will be made available by the authors, without undue reservation.
